# Beyond Organ Boundaries: Molecular Mechanisms of Hepatic Encephalopathy and Parkinson’s Disease from the Perspective of the Gut–Liver–Brain Axis

**DOI:** 10.34133/research.1084

**Published:** 2026-02-06

**Authors:** Tingting Liu, Yuang Ma, Mengdi Zhang, Jianshe Wei

**Affiliations:** ^1^ Institute for Brain Sciences Research, School of Life Sciences, Henan University, Kaifeng 475004, China.; ^2^ College of Pharmacy, Inner Mongolia Medical University, Hohhot, Inner Mongolia 010110, China.; ^3^ Inner Mongolia Key Laboratory of Dairy Biotechnology and Engineering; Key Laboratory of Dairy Products Processing, Ministry of Agriculture and Rural Affairs; Key Laboratory of Dairy Biotechnology and Engineering, Ministry of Education, Inner Mongolia Agricultural University, Hohhot, Inner Mongolia 010018, China.

## Abstract

Hepatic encephalopathy (HE), a severe neurological complication of liver dysfunction, has long been regarded as a clinical issue confined to liver disease. However, recent clinical observations and basic research have revealed complex pathophysiological connections between HE and Parkinson’s disease (PD), 2 seemingly independent conditions. Patients with HE often exhibit irreversible extrapyramidal symptoms that closely resemble the motor disorders of PD; meanwhile, epidemiological studies suggest that individuals with liver disease—particularly non-alcoholic fatty liver disease (NAFLD)—may face an increased risk of developing PD. From the perspective of the gut–liver–brain axis, this study systematically explores the molecular mechanisms linking HE and PD, proposing a core hypothesis: HE creates a unique “neurotoxic soil” through ammonia toxicity, systemic neuroinflammation, and gut–liver–brain axis dysfunction. This soil may trigger PD in susceptible individuals, accelerate subclinical PD progression, or mimic PD-like pathology. The study analyzes in depth the direct regulatory role of ammonia in α-synuclein (α-syn) aggregation, the impact of liver disease-driven neuroinflammation on microglial activation and α-syn propagation, and the hypothesis of liver-derived α-syn transmission via the gut–liver–brain axis. It further discusses synergistic mechanisms such as manganese deposition, neurotransmitter imbalance, and gut microbiota metabolites. Based on these mechanisms, the study prospects translational medical applications, including the development of diagnostic biomarkers and novel therapeutic strategies such as “ammonia clearance plus” and gut–liver–brain axis targeting. This work provides new insights into how environmental metabolic factors contribute to neurodegenerative diseases and offers a theoretical basis for the combined prevention and treatment of HE and PD.

## Introduction: A Seemingly Impossible Connection—From Clinical Observations to Mechanistic Exploration

Hepatic encephalopathy (HE) and Parkinson’s disease (PD) have traditionally been classified into the 2 major disease fields of gastroenterology and neurology, seemingly unrelated. However, clinical observations have found that HE patients often exhibit persistent extrapyramidal symptoms, such as asterixis, muscle rigidity, and bradykinesia, which are highly similar to the motor symptoms of PD [1]. Notably, these motor disorders may persist even after blood ammonia levels return to normal and cognitive function improves. Imaging examinations also indicate abnormal white matter structures in the patient’s basal ganglia, and recent evidence emphasizes that cerebellar functional changes lead to these motor defects in HE. This observation is also newly reported in PD, rather than limited to dysfunction of the basal ganglia. This dual involvement of basal ganglia and cerebellum suggests that HE may constitute a special pathological environment that promotes the occurrence and development of neurodegeneration [2].

As the most prevalent liver disease globally, non-alcoholic fatty liver disease (NAFLD) exhibits the strongest epidemiological association with PD, primarily attributed to its overlap with metabolic syndrome (insulin resistance, obesity) and chronic low-grade inflammation—both key drivers of neurodegeneration. Insulin resistance in NAFLD not only disrupts hepatic glucose metabolism but also impairs insulin signaling in the substantia nigra: Insulin receptors on dopaminergic neurons regulate mitochondrial function and α-synuclein (α-syn) clearance, and their dysfunction directly promotes α-syn aggregation [3,4]. Additionally, NAFLD-related gut microbiota dysbiosis alters metabolite profiles, creating a microenvironment conducive to α-syn aggregation, and the association between NAFLD and PD is dose-dependent—patients with more severe NAFLD [e.g., non-alcoholic steatohepatitis (NASH) or advanced fibrosis] have a higher PD risk than those with milder disease [5]. Alcoholic liver disease (ALD) differs from other liver diseases due to dual neurotoxic insults: direct alcohol metabolites and secondary manganese (Mn) accumulation. Alcohol metabolites can cross the blood–brain barrier (BBB), modify α-syn, and enhance its aggregative and neurotoxic properties [6]. Chronic alcohol consumption also disrupts the gut barrier more severely than other liver diseases, leading to increased entry of gut-derived toxins into the circulation, sustained microglial activation, and subsequent dopaminergic neuron loss [7]. Concurrently, alcohol impairs hepatic Mn excretion, resulting in cerebral Mn accumulation that exacerbates dopaminergic neuron damage. Viral hepatitis [especially hepatitis C virus (HCV) infection] increases PD risk through both direct viral effects and persistent hepatic fibrosis. Viral proteins may cross the BBB, interact with α-syn, and induce its misfolding [8]. Chronic immune activation triggered by viral infection also disrupts protein homeostasis in the central nervous system (CNS). Notably, this association is modifiable: Effective viral clearance reduces PD risk, although patients with preexisting cirrhosis still have a higher risk than healthy controls, highlighting the impact of persistent liver damage [9]. Hepatitis B virus (HBV) shows a weaker correlation with PD, likely due to the lower direct neurotropism of its proteins compared to HCV. Cholestatic liver diseases (e.g., primary biliary cholangitis and primary sclerosing cholangitis) are characterized by bile duct obstruction, which severely impairs hepatic Mn excretion (Mn is primarily eliminated via bile), leading to systemic Mn accumulation [10]. Cerebral Mn deposition (particularly in the globus pallidus) directly inhibits key enzymes involved in dopamine synthesis and induces dopaminergic neuron loss [11]. Accumulated bile acids also disrupt the BBB, facilitating the entry of peripherally aggregated α-syn into the CNS [12].

Despite inconsistencies in conclusions from different studies, severe liver disease and HE are still regarded as an important risk factor for PD or an accelerator of disease progression. Based on the above clues, we propose the “neurotoxic soil” hypothesis: HE creates a diffuse toxic central environment that can interact with PD through 3 pathways—triggering dopaminergic neuron degeneration in genetically susceptible individuals, accelerating pathological processes in the early stages of PD, or simulating PD-like symptoms by disrupting neurotransmitters and basal ganglia function. This hypothesis is based on the combined action of multiple molecular mechanisms. As a core toxic molecule, ammonia, in addition to causing astrocyte edema, can also lead to mitochondrial dysfunction, enhance oxidative stress, and inhibit the protein degradation system, thereby promoting the aggregation and accumulation of misfolded proteins such as α-syn [13]. At the same time, the destruction of the intestinal barrier caused by liver disease allows endotoxins and other substances to enter the circulation, triggering systemic inflammation. These signals enter the CNS through the damaged BBB, leading to the continuous activation of microglia and forming a neuroinflammatory environment similar to that of PD [14]. In addition, according to the Braak staging hypothesis of PD, pathology may originate in the intestine. Liver failure and portosystemic shunts can allow intestinal-derived toxic substances to bypass the liver and directly enter the systemic circulation and nervous system, potentially promoting the spread of pathological proteins into the brain through pathways such as the vagus nerve [15].

**Fig. 1.**
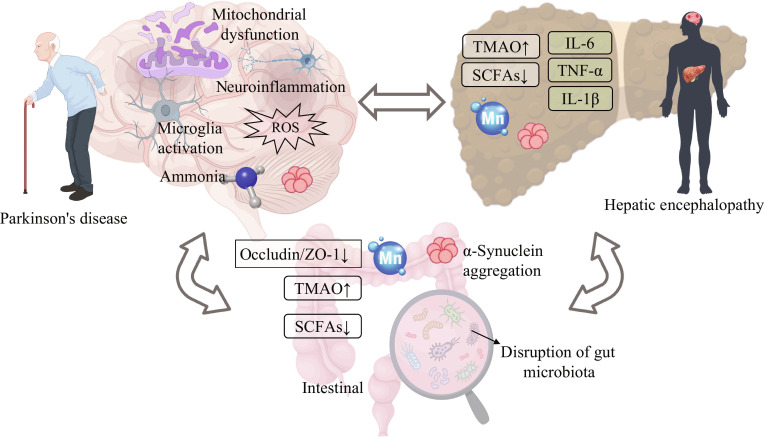
The interaction mechanism among PD, HE, and the intestine. In relation to PD, the brain shows mitochondrial dysfunction, neuroinflammation, microglial activation, production of reactive oxygen species (ROS), and the presence of ammonia. For HE, it involves elevated trimethylamine N-oxide (TMAO), reduced short-chain fatty acids (SCFAs), inflammatory factors such as interleukin-6 (IL-6), tumor necrosis factor-α (TNF-α), interleukin-1β (IL-1β), and the element manganese (Mn). Regarding the intestine, the gut microbiota is disrupted, α-syn aggregates, and occludin/ZO-1 decreases, along with elevated TMAO and reduced SCFAs. In addition, there is a bidirectional interaction between the intestine, the brain, and the liver [10–12].

To sum up, the association between HE and PD goes beyond the similarity of clinical symptoms, revealing the convergence of systemic metabolic diseases and neurodegenerative diseases in underlying mechanisms. Liver disease and HE, through multiple mechanisms such as ammonia neurotoxicity, neuroinflammation, and gut–liver–brain axis disorder, jointly form a “neurotoxic soil” that can induce or accelerate PD pathology. In the future, it is necessary to verify this hypothesis with more precise models and clinical cohorts, and develop intervention strategies targeting these pathways simultaneously, such as ammonia-lowering combined with anti-inflammatory therapy or intestinal flora regulation, to provide new treatment approaches for patients (Fig. 1).

## Ammonia—A Versatile Player Beyond Astrocyte Pathology

**Fig. 2.**
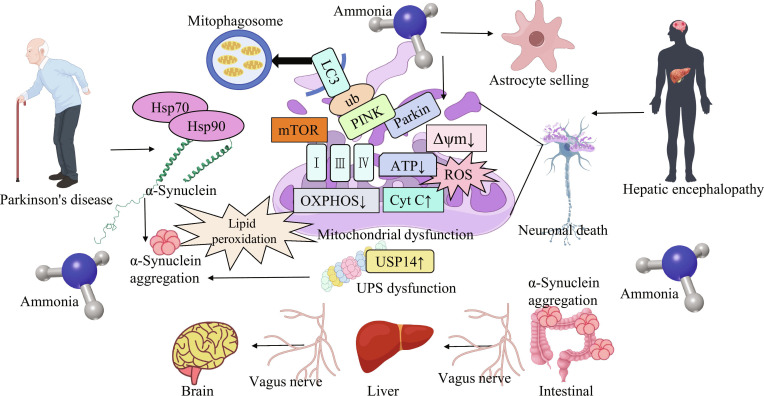
The neurotoxic mechanism of ammonia as the core hub bridging HE and PD. Ammonia exerts effects beyond traditional astrocyte swelling (Alzheimer type II astrocytes) causing acute HE symptoms. It directly disrupts neuronal mitochondria, inhibiting ??OXPHOS, reducing ATP production, and elevating ROS, which drives lipid peroxidation and α-syn aggregation. Ammonia also impairs the UPS by up-regulating USP14 and disrupts mitophagy by interfering with PINK1/Parkin-mediated pathways, further promoting α-syn accumulation. Additionally, the diagram depicts the gut–liver–brain axis, where ammonia and α-syn may propagate through the vagus nerve from the intestine to the liver and then to the brain, contributing to the pathological crosstalk between HE and PD, ultimately leading to neuronal death [36,46,47].

In the study of the pathophysiological mechanisms of HE, ammonia has long been recognized as a core pathogenic factor. Traditional research primarily focuses on its damage to astrocytes and the consequent acute neurological dysfunction, with most evidence derived from acute HE models and in vitro experiments [16,17]. However, when extending the understanding of ammonia’s role to the association between HE and PD—especially considering the potential long-term impact of chronic liver disease on PD pathogenesis—2 critical issues require attention: the applicability of findings from acute models to chronic HE contexts, and the pathophysiological relevance of ammonia concentrations used in existing studies. This section will systematically analyze the neurotoxic mechanisms of ammonia from both traditional and novel perspectives while critically discussing the limitations of current research models and concentration settings, with a specific focus on the association between ammonia and α-syn abnormalities, mitochondrial dysfunction, and protein degradation system impairment (Fig. 2).

### Limitations of traditional ammonia neurotoxicity research: Dominance of acute models and poor adaptability to chronic HE

Traditional research on ammonia’s neurotoxicity centers on its induction of astrocyte swelling (forming Alzheimer type II astrocytes) and the subsequent development of acute HE symptoms (e.g., cognitive disturbance and altered consciousness). For instance, ammonia disrupts astrocyte homeostasis by impairing glutamate–glutamine cycling and inducing osmotic stress, leading to astrocyte edema and brain edema in acute HE [13]. While these findings effectively explain the acute neurological deterioration in patients with severe liver failure (e.g., fulminant hepatic failure), they have limitations when applied to chronic HE—a condition commonly associated with cirrhosis and a key risk factor for PD as highlighted by epidemiological studies [18].

Chronic HE is characterized by long-term, low-level ammonia exposure (blood ammonia levels typically 50 to 150 μM in stable cirrhotic patients, compared to 100 to 200 μM in acute HE) [19], rather than the sudden, high ammonia spikes observed in acute HE. In this context, ammonia’s neurotoxicity may manifest as cumulative, subtle damage (e.g., gradual mitochondrial dysfunction and progressive α-syn aggregation) rather than acute astrocyte swelling. However, existing literature on ammonia’s effects is dominated by acute models (e.g., acute ammonia loading in rodents and fulminant liver failure models) and in vitro studies, which fail to replicate the chronic, low-concentration ammonia environment of clinical chronic HE. This mismatch raises questions about whether the mechanisms identified in acute models (e.g., acute astrocyte damage) can fully explain ammonia’s role in promoting long-term neurodegenerative processes (e.g., PD-like pathology) in chronic liver disease patients.

### Beyond astrocyte pathology: Ammonia damages neuronal mitochondria to drive α-syn aggregation

Mitochondria, as the “powerhouses” of cells, are crucial for high-energy-consuming neurons, and their dysfunction is one of the core mechanisms in PD pathogenesis. Ammonia can directly damage the structure and function of neuronal mitochondria, triggering energy metabolism disorders and intensified oxidative stress, thereby creating pathological conditions for the abnormal aggregation of α-syn [20].

In terms of energy metabolism impairment, ammonia reduces adenosine triphosphate (ATP) production by inhibiting the activity of mitochondrial respiratory chain complexes. In vitro experiments have shown that after treating primary rat substantia nigra dopaminergic neurons with ammonia for 24 h, the activities of complexes I, III, and IV decrease, and ATP levels reduce [21]; the activity of complex I and ATP levels in the substantia nigra of portosystemic shunt rats also decrease, and the degree of damage is positively correlated with brain ammonia concentration [22]. The decrease in complex I activity not only leads to energy deficiency but also disrupts the mitochondrial membrane potential, promoting the opening of mitochondrial permeability transition pores and the release of cytochrome C (Cyt C) to activate the apoptosis pathway, exacerbating neuronal death [23]. Meanwhile, ATP deficiency affects the correct folding of α-syn and its binding to synaptic vesicles, making it more likely to convert from soluble monomers to insoluble aggregates [24].

Ammonia also exacerbates oxidative stress through mitochondrial dysfunction, which is a key driver of α-syn aggregation [25]. Impaired respiratory chain complexes generate large amounts of reactive oxygen species (ROS) such as superoxide anions, and ammonia inhibits the activity of antioxidant enzymes like glutathione peroxidase, reducing the ability to scavenge ROS [26]. In in vitro experiments, after ammonia treatment, the ROS level in neurons increases, and lipid peroxidation products and protein oxidation products increase, while ROS scavengers can alleviate the damage [27]. ROS can oxidize the cysteine residues of α-syn to change its conformation, and lipid peroxidation products [such as 4-hydroxynonenal (4-HNE)] can also bind to the lysine residues of α-syn to enhance aggregation. The content of α-syn oligomers in the substantia nigra of HE model mice increases, and after antioxidant treatment, their content decreases, and neuronal loss is reduced, confirming the mechanism by which ammonia promotes abnormal α-syn aggregation through the mitochondrial dysfunction–oxidative stress axis [28].

In addition, ammonia-induced mitochondrial dysfunction also affects the process of mitophagy, further aggravating the toxic effects of α-syn [29]. Ammonia inhibits the mitochondrial translocation of the mitophagy regulatory factor Parkin, leading to the accumulation of damaged mitochondria and α-syn. The mitochondrial localization level of Parkin in the substantia nigra of portosystemic shunt rats decreases, and the ratio of autophagy marker LC3-II/LC3-I decreases. After overexpressing Parkin, α-syn aggregates decrease, and neuronal survival increases [30]. In summary, through 3 dimensions—energy metabolism impairment, intensified oxidative stress, and inhibition of mitophagy—ammonia induces mitochondrial dysfunction to promote abnormal α-syn aggregation, which not only explains the extrapyramidal symptoms in HE patients but also provides molecular evidence for the pathological association between HE and PD.

### Novel perspective: Direct neurotoxic effects of ammonia and α-syn

The abnormal aggregation of α-syn is the core pathological feature of PD. Traditionally, it was believed that ammonia participates in the pathogenesis of HE only by causing astrocyte swelling. However, recent studies have found that ammonia can transcend cell type limitations and directly act on the α-syn metabolic pathway within neurons. By interfering with mitochondrial function, inducing protein misfolding, and inhibiting proteasome activity, it promotes the pathological transformation of α-syn, thus becoming a key molecular bridge connecting HE and PD [31].

Firstly, ammonia creates a favorable environment for the abnormal aggregation of α-syn by inducing mitochondrial dysfunction. As the core organelle maintaining neuronal energy homeostasis and redox balance, the functional state of mitochondria directly affects the folding stability and cytotoxicity of α-syn [32]. Ammonia can interfere with the mitochondrial respiratory chain complexes and matrix enzyme systems, specifically by inhibiting the activities of complexes I and IV, hindering the process of oxidative phosphorylation, and leading to a reduction in ATP production [33]. Energy deficiency further impairs the ability of molecular chaperones such as heat shock protein 70 (Hsp70) and Hsp90 to assist in the correct folding of α-syn. At the same time, it affects synaptic vesicle recycling, causing α-syn to accumulate at the presynaptic membrane and initiate aggregation [34]. In addition, ammonia induces mitochondria to generate a large amount of ROS and inhibits the activity of antioxidant enzymes, resulting in ROS accumulation. Excessive ROS causes tyrosine nitration and cysteine oxidation of α-syn, changing its secondary structure from α-helix to β-folding, thereby accelerating the formation of oligomers [35]. On the other hand, ammonia also regulates the mammalian target of rapamycin (mTOR) signaling pathway and reduces the mitochondrial membrane potential, inhibiting the expression of PINK1 and the mitochondrial localization of Parkin, thereby impairing mitophagy function [36]. This not only leads to the accumulation of damaged mitochondria, further exacerbating the energy crisis and oxidative stress, but also promotes the release of Cyt C and its binding with α-syn to form a complex, enhancing the aggregation ability of α-syn and inhibiting apoptosis, allowing toxic α-syn to continuously accumulate within neurons.

In addition to indirectly acting through the mitochondrial pathway, ammonia has also been proven to directly promote the misfolding and aggregation of α-syn. The pathological transformation of α-syn is a process in which soluble monomers gradually form toxic oligomers and even insoluble fibrillar aggregates [37]. In the past, it was believed that this process mainly originated from genetic mutations or age-related protein homeostasis imbalance. However, recent studies have found that ammonia can act directly on α-syn molecules as an exogenous inducer. In a cell-free system, ammonia affects the protonation state of α-syn amino residues, disrupting the endogenous salt bridge between its N-terminal lysine residues and C-terminal acidic amino acids, causing the protein structure to change from a folded state to a disordered state, exposing the hydrophobic core, and thereby promoting the formation of oligomers between monomers through hydrophobic interactions [38]. In addition, ammonia can also enhance the β-sheet tendency of α-syn, accelerate the formation of fibrillar aggregates, and make the finally formed aggregates have a larger diameter and more branches [39]. These α-syn oligomers induced by ammonia exhibit stronger neurotoxicity and can cause damages such as shortening of neuronal processes and decrease in mitochondrial membrane potential [40]. In animal models, both acute and chronic ammonia loading can cause a substantial increase in α-syn oligomers and fibrillar aggregates in the substantia nigra and striatum of rats, accompanied by behavioral abnormalities such as bradykinesia and muscle rigidity. These symptoms can be partially alleviated by α-syn antibodies, suggesting a direct correlation with α-syn aggregation [41]. Analysis of brain tissue from patients with cirrhotic HE also found the presence of Lewy body-like α-syn aggregation in the substantia nigra, and the degree of aggregation was positively correlated with the patients’ blood ammonia levels and clinical symptoms [42]. In addition, α-syn-positive particles were also detected in peripheral vagal ganglia, indicating that ammonia may simultaneously promote α-syn pathological changes in the central and peripheral nervous systems, which provides a basis for the transmission of α-syn mediated by the gut–liver–brain axis [43].

Ammonia can also impair the cell’s ability to clear misfolded α-syn by inhibiting the function of the ubiquitin–proteasome system (UPS). As a key pathway for degrading abnormal proteins, UPS can be directly inhibited by ammonia in terms of the catalytic activity of the 20*S* core particle in the 26*S* proteasome. On one hand, ammonia interferes with substrate degradation by competitively binding to the active site; on the other hand, it alters the intracellular pH, causing conformational changes in the proteasome and thereby reducing substrate entry [44]. In neurons, the inhibitory effect of ammonia on the proteasome is particularly significant, leading to delayed clearance of misfolded α-syn, a significant prolongation of its half-life, and accumulation of ubiquitinated α-syn [45]. At the same time, ammonia disrupts the function of the ubiquitinating enzyme system, including inhibiting the activity of E1-activating enzyme and down-regulating the expression of E3 ligase Parkin, which reduces the ubiquitination labeling of α-syn and hinders its targeted degradation [46]. More notably, ammonia can activate deubiquitinating enzymes such as ubiquitin-specific protease 14 (USP14), further removing ubiquitin chains from α-syn and converting it from a degradable state to a nondegradable state [47]. In the brain tissue of HE patients, the increased activity of USP14 in the substantia nigra is closely related to the degree of α-syn aggregation.

There is a vicious cycle between ammonia-induced UPS dysfunction and α-syn aggregation: Decreased UPS function leads to α-syn accumulation, while α-syn oligomers and fibrillar aggregates, in turn, can inhibit proteasome activity and competitively interfere with the degradation of other misfolded proteins, ultimately triggering widespread collapse of protein homeostasis [48]. This cyclic mechanism may explain why extrapyramidal symptoms in some HE patients remain difficult to fully reverse even after blood ammonia levels are controlled—long-term UPS dysfunction and α-syn aggregation have formed a self-sustaining pathological state. Even if the toxic factor of ammonia is removed, the “inertia” of α-syn aggregation will continue to drive the neurodegenerative process [49]. In summary, ammonia directly promotes the pathological transformation of α-syn through multiple molecular mechanisms, including disrupting mitochondrial function, directly inducing protein misfolding, and inhibiting proteasome degradation capacity, thereby establishing an important pathological link between HE and PD.

### Chronic hyperammonemia and neuroinflammation: A sustained driver of PD-like progression

In addition to pathological changes in α-syn, the persistent presence of chronic hyperammonemia in chronic HE also promotes low-grade but persistent neuroinflammation, which is the core driving factor for progressive neurodegenerative changes in PD. Unlike acute HE (where neuroinflammation is triggered by sudden endotoxemia and BBB disruption), chronic hyperammonemia induces a sustained proinflammatory state in the brain that amplifies PD-related pathology. In chronic HE models, long-term low-level ammonia exposure primes microglia in the substantia nigra, increasing their responsiveness to secondary triggers [e.g., gut-derived lipopolysaccharide (LPS)] [50]. Primed microglia exhibit enhanced secretion of tumor necrosis factor-α (TNF-α) and interleukin-1β (IL-1β), accelerating dopaminergic neuron loss when combined with α-syn aggregates—mirroring PD’s “dual-hit” pathogenesis (environmental triggers and neuroinflammation). Notably, chronic hyperammonemia also impairs cerebellar synaptic transmission and Purkinje cell function in HE models, leading to motor coordination deficits that overlap with cerebellum-mediated motor symptoms in PD; this extends the pathological link between HE and PD beyond basal ganglia dysfunction to include cerebellar impairment [51,52]. Chronic ammonia exposure also impairs astrocyte function, reducing the production of neurotrophic factors and increasing the release of proinflammatory cytokines [53], creating a neurotoxic microenvironment that promotes α-syn propagation. Post-mortem studies of cirrhotic patients with chronic HE further confirm increased activated microglia and astrocyte dysfunction in the substantia nigra, with the degree of neuroinflammation positively correlated with PD-like motor symptom severity [54].

While ammonia is a well-recognized core toxin in HE, emerging evidence confirms subtle ammonia dysregulation in PD (though less severe than in HE). Epidemiological studies show that PD patients with comorbid liver dysfunction have higher plasma ammonia levels than PD patients with normal liver function [55]. In 1-methyl-4-phenyl-1,2,3,6-tetrahydropyridine (MPTP)-induced PD mouse models, brain ammonia concentrations increase compared to controls, attributed to reduced activity of glutamine synthetase in astrocytes [56]. Overactivity of glutamatergic projection neurons and beneficial effect of antiglutamatergic substances in animal experiments suggest that excess supply of glutamate might contribute to the pathophysiology of PD. Reduced activity of the glutamate-metabolizing enzyme glutamine synthetase leads to decreased uptake of glutamate and thus abundant glutamate [57]. Importantly, mild hyperammonemia in PD exacerbates existing pathological processes, enhances microglial activation through the Toll-like receptor 4 (TLR4)/nuclear factor κB (NF-κB) pathway, and increases the neurotoxicity of α-syn oligomers on dopaminergic neurons [58], consistent with the “neurotoxic soil” hypothesis for HE-PD association. Together, these findings from chronic hyperammonemia research underscore a critical point: Sustained low-level ammonia exposure in chronic HE creates a “neurotoxic soil” that drives the definition of progressive pathological changes in PD (α-syn aggregation, neuroinflammation, dopaminergic loss), filling the gap left by acute ammonia models in explaining the association between HE-PD.

## Neuroinflammation: How Liver Disease-Driven Systemic “Fire” Ignites the Brain

### Systemic inflammation in liver diseases: Intestinal barrier damage and peripheral inflammation

In the pathological association between HE and PD, neuroinflammation is not an independent process but an extension of peripheral inflammatory responses (driven by intestinal barrier dysfunction) to the CNS—this shared inflammatory cascade is the key link between the 2 pathologies. The intestine and liver maintain homeostasis via the gut–liver axis under physiological conditions: Gut microbiota metabolites enter the liver via the portal vein for metabolism, while the liver regulates gut barrier function and microbiota composition through bile acid secretion and antibacterial substance synthesis, preventing pathogen-associated molecular patterns (PAMPs) such as LPS from entering systemic circulation [59].

When intestinal homeostasis is disrupted (a feature common to both HE and PD), the intestinal barrier—comprising physical, chemical, immune, and biological barriers—sustains structural and functional damage, becoming the “first crack” triggering peripheral inflammation. Physically, tight junction proteins [occludin, zona occludens 1 (ZO-1)] between intestinal epithelial cells are down-regulated, expanding intercellular gaps and allowing macromolecules like LPS to penetrate the lamina propria [60]; chemically, reduced mucin secretion thins the mucus layer, and decreased bile acid synthesis impairs antibacterial defense [61]; immunologically, intestinal immune cell phagocytic capacity declines, and T cell subsets imbalance, weakening PAMP clearance [62]; biologically, gut dysbiosis (reduced beneficial bacteria, increased Gram-negative bacteria) elevates LPS production, further exacerbating barrier damage [63].

Clinically, intestinal biopsies of HE patients confirm reduced tight junction protein expression (correlated with blood ammonia levels) and increased epithelial apoptosis, while PD patients also exhibit similar intestinal barrier impairment and dysbiosis [64]. Once the barrier is compromised, LPS and other PAMPs enter systemic circulation, binding to TLR4 on peripheral immune cells to activate the myeloid differentiation primary response protein 88 (MyD88)–NF-κB/mitogen-activated protein kinase (MAPK) pathway, releasing proinflammatory factors (IL-1β, TNF-α, IL-6) [65,66]. This chronic low-grade peripheral inflammation (shared by HE and PD) lays the foundation for inflammatory signal transmission to the CNS, rather than liver damage itself—liver dysfunction in HE primarily exacerbates intestinal barrier disruption, while PD-related intestinal alterations independently trigger analogous peripheral inflammation.

### Sustained activation of microglia: From peripheral signals to central neurotoxicity

In liver disease/PD-related neuroinflammation, sustained microglial activation bridges peripheral inflammation and central neurotoxicity—a mechanism common to both pathologies. Under healthy conditions, microglia exist in a surveillance state; peripheral inflammatory signals (LPS, proinflammatory cytokines) induce neuroinflammation not only by crossing the BBB or transmitting via the vagus nerve but also through infiltration of immune system cells [67]. Peripheral immune cells (e.g., monocytes, T lymphocytes, and neutrophils) activated by systemic inflammation can adhere to and migrate across the compromised BBB (a hallmark of both HE and PD), infiltrating brain parenchyma, particularly in vulnerable regions like the substantia nigra [68]. These infiltrating immune cells release proinflammatory cytokines and ROS locally, directly activating resident microglia and amplifying neuroinflammation—a synergistic effect that accelerates dopaminergic neuron damage more potently than microglial activation alone.

Peripheral inflammatory signals cross the BBB to affect microglia mainly through 2 pathways. The first is the BBB pathway: In states of liver cirrhosis or HE, peripheral inflammatory factors such as TNF-α and IL-1β can disrupt the tight junctions between brain microvascular endothelial cells, and hyperammonemia also inhibits the synthesis of tight junction proteins, resulting in increased BBB permeability [69]. This allows PAMPs such as LPS and peripheral immune cells to easily enter the brain parenchyma, accumulating particularly in regions such as the substantia nigra and hypothalamus, directly activating microglia [70]. The second is the neurohumoral pathway: Intestinal-derived inflammatory signals can be transmitted to the nucleus tractus solitarius of the brainstem through afferent fibers of the vagus nerve, thereby affecting other brain regions [71]; cytokines can also enter the CNS through specific transporters on the BBB or induce surrounding cells to release secondary inflammatory signals, indirectly promoting microglial activation [72].

Once activated, microglia undergo significant phenotypic transformation, with the TLR4–NF-κB signaling pathway at the core of this mechanism. After LPS binds to TLR4 on the cell surface, a MyD88-dependent cascade reaction of myeloid differentiation factors ultimately leads to the translocation of NF-κB into the nucleus, initiating the transcription of proinflammatory factors such as IL-1β, TNF-α, and IL-6, as well as enzymes including inducible nitric oxide synthase (iNOS) and cyclooxygenase-2 (COX-2) [73]. These factors damage dopaminergic neurons through various pathways: for example, causing mitochondrial dysfunction, inducing apoptosis, and altering the Bax/Bcl-2 balance [74]. Meanwhile, activated microglia produce ROS and reactive nitrogen species (RNS), triggering oxidative stress, impairing tyrosine hydroxylase activity and cellular structures, and releasing matrix metalloproteinases (MMPs) and complement components, which further disrupt the neuronal microenvironment and form a persistent neurotoxic state [75].

Particularly importantly, recent studies have revealed that microglia not only passively respond to inflammation but also actively contribute to the progressive deterioration of the disease—yet a critical paradox exists: Despite their phagocytic capacity, activated microglia in HE/PD pathologies fail to effectively clear aggregated α-syn and even promote its pathological spread [76]. Microglia participate in the pathological spread of α-syn through a “phagocytosis–release” cycle: Cell surface receptors (e.g., TREM2 and CD36) recognize and phagocytose α-syn aggregates [77], but in the context of chronic neuroinflammation (driven by liver disease), lysosomal dysfunction and pyroptosis in activated microglia prevent complete degradation of internalized α-syn [78]. Instead, these toxic aggregates are re-released extracellularly (via exocytosis or cell lysis), thereby infecting surrounding healthy neurons [48]. Additionally, proinflammatory cytokines (e.g., TNF-α and IL-1β) precondition neurons to be more sensitive to α-syn, further promoting pathological diffusion [79].

The failure of activated microglia to clear α-syn aggregates stems from multiple interconnected mechanisms: (a) Chronic exposure to ammonia and LPS impairs lysosomal acidification and reduces the activity of lysosomal hydrolases in microglia, directly compromising their ability to degrade phagocytosed α-syn [80]. (b) Activated microglia shift toward a proinflammatory (M1) phenotype, prioritizing the secretion of proinflammatory factors over phagocytic function—this phenotypic skewing is exacerbated by liver disease-driven systemic inflammation, which up-regulates TLR4/NF-κB signaling and suppresses anti-inflammatory (M2) polarization (e.g., via reduced IL-4/IL-10 signaling) [81]. (c) α-Syn aggregates undergo posttranslational modifications (e.g., phosphorylation and glycation) in the HE/PD microenvironment, which mask phagocytic recognition motifs and reduce their uptake by microglia [82]. (d) The neurotoxic microenvironment (high ROS, mitochondrial dysfunction) in HE/PD impairs microglial viability and phagocytic efficiency, creating a vicious cycle where impaired microglia fail to clear α-syn, and accumulated α-syn further exacerbates microglial activation and dysfunction [58,82,83].

**Fig. 3.**
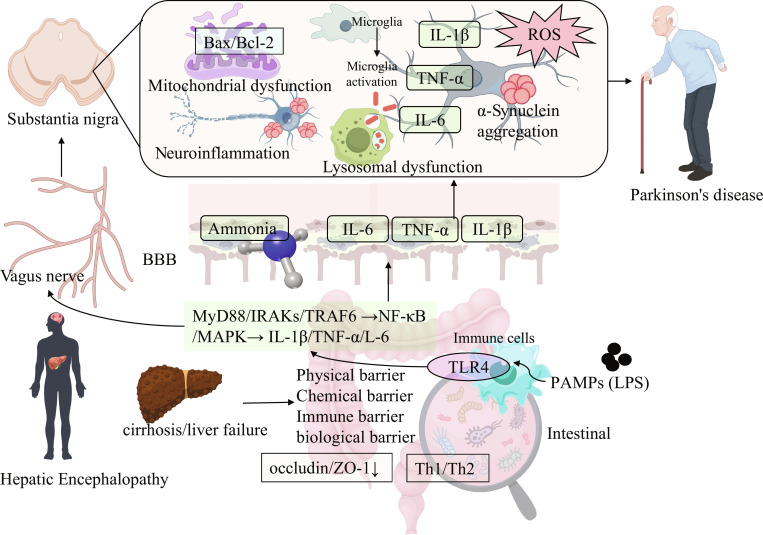
The systemic inflammation driven by liver disease triggers a cascade reaction of neuroinflammation bridging HE and PD. Liver cirrhosis or failure disrupts intestinal barriers (physical, chemical, immune, and biological barriers, with reduced expression of tight junction proteins like occludin/ZO-1, causing “leaky gut”). PAMPs (e.g., LPS) enter the bloodstream, triggering a systemic inflammatory response. Through TLR4 on immune cells, this activates signaling cascades (MyD88/IRAKs/TRAF6 → NF-κB/MAPK), leading to the release of proinflammatory cytokines (IL-1β, TNF-α, IL-6). These peripheral inflammatory signals, along with ammonia, cross the BBB. In the brain (substantia nigra), they activate microglia. Activated microglia, combined with factors like α-syn aggregation, ROS, mitochondrial dysfunction (involving Bax/Bcl-2 pathways), and lysosomal dysfunction, create a neurotoxic central environment. This environment promotes further neuroinflammation and neurodegeneration, contributing to PD pathology [65,66,81].

In summary, systemic inflammation induced by intestinal barrier dysfunction (shared by HE and PD) enters the brain through multiple pathways, including BBB disruption, vagal signal transmission, and immune cell infiltration—leading to the sustained activation of microglia. This not only directly contributes to the formation of a neurotoxic environment but also actively drives the progression of PD pathology in HE by promoting α-syn spread and inhibiting neurogenesis. This mechanism provides an important perspective for understanding the intrinsic connection between the 2 diseases and points out the direction for developing therapeutic strategies targeting microglia and neuroinflammatory processes (Fig. 3).

## The Gut–Liver–Brain Axis and the Hypothesis of “Hepatogenic” Transmission of α-Syn

**Fig. 4.**
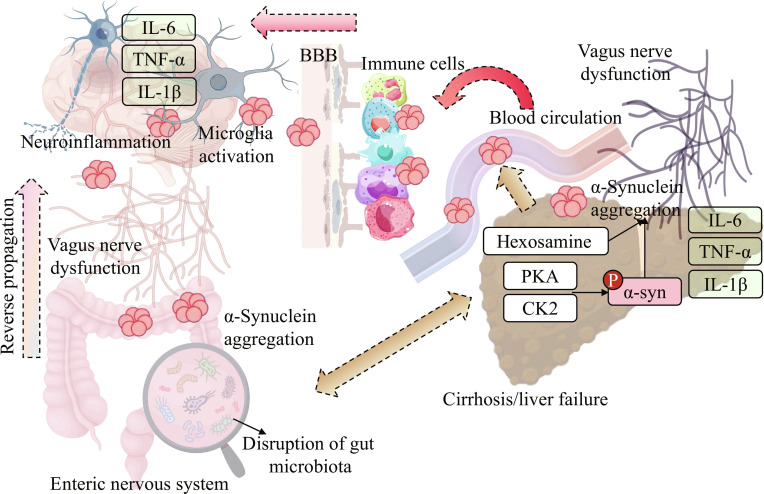
The “hepatogenic” propagation mechanism of α-syn under the disorder of the gut–liver–brain axis. Disruption of the gut microbiota leads to α-syn aggregation in the ENS. Meanwhile, in the state of cirrhosis or liver failure, the liver modifies α-syn through phosphorylation via PKA (a ubiquitously expressed kinase, not liver-specific), CK2, etc., enhancing its aggregability, and releases inflammatory factors such as IL-6, TNF-α, and IL-1β. Vagus nerve dysfunction not only accelerates the retrograde propagation of α-syn from the gut to the brain but also facilitates the entry of liver-derived α-syn into the CNS through the blood circulation, immune cells, and other pathways across the BBB. The α-syn and inflammatory factors entering the CNS activate microglia, trigger neuroinflammation, and ultimately promote the pathological progression related to PD, while the imbalance of the gut–liver–brain axis in the state of HE is a key hub in this process [115,116,137].

The gut–liver–brain axis, as a complex regulatory network connecting the gut, liver, and CNS, plays a crucial role in maintaining metabolic balance and neurological stability. In recent years, with the proposal and refinement of the “gut-origin hypothesis” (Braak hypothesis) for PD, the role of the gut–liver–brain axis in the pathological progression of PD has gradually garnered attention [84]. Under HE conditions, impaired liver function and disrupted gut microbiota disrupt the homeostasis of the gut–liver–brain axis. This not only directly leads to neurological abnormalities but may also serve as a key pathological link between HE and PD by regulating the peripheral origin and central transmission of α-syn [85]. This section will extend the Braak hypothesis to systematically explore how disruptions in the gut–liver–brain axis accelerate the “hepatogenic” transmission of α-syn through 3 dimensions: “failure of the liver barrier”, “hepatic processing of α-syn”, and “vagus nerve dysfunction”. This provides a novel systems biology perspective on the association between HE and PD (Fig. 4).

### Extension of the Braak hypothesis: PD pathology originates in the ENS, with α-syn propagating retrogradely along the vagus nerve

In 2003, based on pathological staging studies of brain tissues from PD patients, Braak and colleagues [86] proposed the renowned “Braak hypothesis”: The pathological process of PD does not originate in the CNS but rather begins in the enteric nervous system (ENS) or olfactory mucosa. Subsequently, abnormally aggregated α-syn propagates retrogradely through neural pathways to the CNS, ultimately affecting dopaminergic neurons in the substantia nigra and triggering clinical symptoms [87]. This hypothesis overturned the traditional “central origin” perspective, providing a new direction for understanding the early pathogenesis of PD and peripheral interventions. In recent years, both basic and clinical research have continually provided supporting evidence for this hypothesis.

From the perspective of pathological transmission pathways, the ENS, as the largest peripheral autonomic nervous system, forms close neural connections with the CNS via the vagus nerve, providing an anatomical basis for the retrograde propagation of α-syn. The ENS consists of the myenteric plexus and submucosal plexus, containing a vast number of neurons (approximately 500 million, comparable to the number of spinal cord neurons) and glial cells. It independently regulates intestinal motility, secretion, and absorption functions while communicating with the CNS through afferent fibers of the vagus nerve (vagal nodose ganglion → brainstem solitary tract nucleus) [88]. Studies have found that in the early pathological stages of PD patients (Braak stages 1 and 2), α-syn aggregates (Lewy body-like structures) already appear in the ENS, and the distribution of these aggregates highly coincides with the intestinal innervation regions of the vagus nerve afferent fibers [89]. Simultaneously, α-syn aggregates have been detected in the vagal nodose ganglion and the brainstem solitary tract nucleus, while central nuclei such as the substantia nigra remain unaffected. This suggests that α-syn may propagate retrogradely from the ENS to the CNS along the vagus nerve afferent fibers [90].

Animal experiments have further confirmed the gut-to-central transmission pathway of α-syn. After injecting preformed α-syn fibrils (PFFs) into the intestinal muscle layer of mice, researchers observed through immunofluorescence staining that α-syn aggregates first appeared in ENS neurons at the injection site (1 month), gradually spreading to the vagal nodose ganglion (2 months), the brainstem solitary tract nucleus (3 months), the locus coeruleus (4 months), and finally detecting α-syn aggregates in the substantia nigra (6 months). This propagation process was accompanied by degenerative damage to the vagus nerve afferent fibers [91]. Critically, α-syn aggregates were also identified in other brain regions including the hippocampus, striatum, and cerebellum—regions closely linked to nonmotor (cognitive, emotional) and motor symptoms of PD—rather than being restricted to the substantia nigra [92]. However, when the vagus nerve was severed, the central transmission of α-syn was completely blocked, and no α-syn aggregates appeared in any brain region (including the substantia nigra), confirming that the vagus nerve is a critical pathway for the gut-to-central transmission of α-syn [93]. Additionally, α-syn aggregates that propagate to the CNS exhibit a “seeding effect”—they can induce misfolding and aggregation of endogenous α-syn, forming new aggregates that further spread, ultimately leading to the degeneration and death of dopaminergic neurons in the substantia nigra and the emergence of PD-like motor symptoms (such as bradykinesia and resting tremor) [94].

Clinically, post-mortem analyses of PD patients show that α-syn pathology spreads in a stereotypical sequence across brain regions: starting in the brainstem (solitary tract nucleus, locus coeruleus) in early stages, then progressing to the substantia nigra, striatum, and eventually cortical regions (frontal, temporal, hippocampal) in advanced stages [95]. In preclinical models, peripheral (intestinal/hepatic) α-syn PFFs injected into rodents not only accumulate in the substantia nigra but also infiltrate brain regions including the ventral tegmental area, amygdala, and prefrontal cortex—consistent with the clinical progression of PD pathology [96]. The apparent enrichment of α-syn in the substantia nigra reflects the selective vulnerability of dopaminergic neurons (high metabolic demand, sensitivity to oxidative stress) to α-syn-induced toxicity, rather than a lack of α-syn entry into other brain areas.

The mechanism of retrograde propagation of α-syn may be related to “template induction” and “neurotransmitter transport.” On the one hand, exogenous α-syn fibrils (such as gut-derived PFFs) can act as “seeds”, binding to soluble α-syn monomers within neurons and inducing conformational changes to form new aggregates. This process is similar to the “conformational propagation” of prions [94]. On the other hand, α-syn can transfer between neurons via synaptic vesicle transport mechanisms—α-syn binds to phospholipids (such as phosphatidylserine) on synaptic vesicle membranes and transfers between pre- and post-synaptic neurons through the release and uptake of synaptic vesicles. The dense synaptic connections of vagus nerve afferent fibers provide favorable conditions for this transfer process [97]. Furthermore, impaired intestinal barrier function may exacerbate the peripheral origin of α-syn. Gut microbiota metabolites (such as LPS) and intestinal pathogens (such as *Helicobacter pylori*) can disrupt the intestinal epithelial barrier, allowing α-syn in the gut (such as α-syn homologous proteins produced by gut bacteria or abnormally secreted α-syn by intestinal epithelial cells) to enter the intestinal wall. Once taken up by ENS neurons, this can trigger aggregation, serving as the “initial trigger” for the gut origin of α-syn [98].

The extended significance of the Braak hypothesis lies in its expansion of the pathological origin of PD from the CNS to the peripheral gut, providing an important theoretical link between HE and PD. Under HE conditions, disruptions in gut microbiota, impaired intestinal barrier function, and reduced liver detoxification may exacerbate the production, aggregation, and peripheral circulation of α-syn. This, in turn, accelerates its propagation to the CNS via the gut–liver–brain axis, serving as a key mechanism for the occurrence of PD-like symptoms in HE patients and the increased risk of PD onset.

### How does liver disease accelerate this process?

As the central hub of the gut–liver–brain axis, the liver serves dual functions as both a “detoxifier of gut-derived toxins” and a “metabolic regulation center”. When liver function is impaired (e.g., due to cirrhosis or liver failure), its ability to clear neurotoxic substances of intestinal origin, including α-syn, is compromised. Simultaneously, the liver may modify α-syn through its own metabolic activities. Combined with vagus nerve dysfunction, these factors collectively accelerate the gut-to-central transmission of α-syn. This process can be summarized as a triple mechanism: “failure of the barrier–processing and modification–pathway abnormalities”.

#### Failure of the liver as the “first line of defense”

Under normal physiological conditions, the liver serves as the “first line of defense” for clearing neurotoxic substances of intestinal origin. Gut bacterial metabolites (such as ammonia and LPS) and abnormal proteins secreted by intestinal cells (such as α-syn) enter the liver via the portal vein. They are then degraded or inactivated by the detoxification enzyme systems of hepatocytes (e.g., cytochrome P450 enzymes and glutathione transferases) and subsequently excreted into bile or converted into harmless substances that enter the systemic circulation. This process prevents these toxins from reaching the CNS [99]. However, in liver diseases (such as cirrhosis or severe hepatitis), this defense mechanism fails due to reduced liver function and the development of portosystemic shunts. As a result, neurotoxic substances of intestinal origin, including modified α-syn, directly enter the systemic circulation, creating conditions for their transmission to multiple brain regions [100].

The loss of detoxification capacity due to impaired liver function is the core reason for the failure of this defense. In cirrhotic patients, extensive necrosis of hepatocytes and disruption of the liver lobule structure significantly reduce the activity of detoxification enzyme systems, diminishing the liver’s ability to clear gut-derived α-syn [101]. In vitro experiments have confirmed that normal hepatocytes can phagocytose gut-derived α-syn oligomers and degrade them in lysosomes. In contrast, hepatocytes from cirrhotic patients exhibit reduced phagocytic capacity for α-syn and decreased lysosomal enzyme activity, leading to the accumulation of α-syn within hepatocytes. This accumulated α-syn is subsequently released into the portal vein via hepatocyte apoptosis and enters the systemic circulation [102]. Additionally, impaired liver function reduces the synthesis of plasma proteins (such as albumin), which can bind to α-syn and inhibit its aggregation and neurotoxicity [103]. Lower plasma albumin levels in cirrhotic patients result in increased levels of free α-syn, further elevating the risk of its entry into multiple brain regions [104].

The development of portosystemic shunts is another critical factor in the failure of the liver’s defense. In cirrhotic patients, elevated portal venous pressure leads to the formation of portosystemic shunts (including spontaneous and artificial shunts), allowing portal blood to bypass the liver and directly enter the systemic circulation (e.g., the vena cava). This completely bypasses the liver’s detoxification function [105]. In a rat model with portosystemic shunts, the injection of isotopically labeled α-syn oligomers into the intestine resulted in higher concentrations of α-syn in the systemic circulation compared to normal rats. Furthermore, the amount of α-syn entering the CNS increased, along with elevated levels of α-syn aggregates in the substant nigra, striatum, and hippocampus and greater loss of dopaminergic neurons. This confirms that portosystemic shunts significantly promote the central transmission of gut-derived α-syn [106]. Clinical studies have also found that cirrhotic patients with portosystemic shunts have a significantly higher incidence of PD-like symptoms (such as bradykinesia and muscle rigidity) compared to those without shunts, along with significantly worse motor function scores. This suggests a close correlation between portosystemic shunts and α-syn transmission, as well as PD-like symptoms [107].

In addition to α-syn, the failure of the liver’s defense allows other neurotoxic substances of intestinal origin [such as ammonia, LPS, and short-chain fatty acids (SCFAs)] to enter the systemic circulation. These substances can “pave the way” for the central transmission of α-syn by disrupting the BBB and activating neuroinflammation. For example, LPS can activate microglia to release inflammatory cytokines, increasing the permeability of the BBB and making it easier for peripherally circulating α-syn to enter the CNS. Simultaneously, inflammatory cytokines can induce misfolding of α-syn in central neurons, enhancing its “seeding effect” [108]. In cirrhotic patients, elevated plasma LPS levels are positively correlated with the content of α-syn aggregates in the substantia nigra, confirming the synergistic effect of gut-derived neurotoxins and α-syn transmission [109].

#### The liver as a “processing factory” for α-syn

Traditionally, α-syn was thought to be primarily expressed in the CNS and peripheral nervous system (e.g., ENS). However, recent studies have revealed that the liver, as a key metabolic organ, not only takes up peripherally derived α-syn but also may produce α-syn itself. Through posttranslational modifications (e.g., phosphorylation, glycation, and ubiquitination), the liver can alter the aggregation propensity and neurotoxicity of α-syn, effectively acting as a “peripheral processing factory” for α-syn. This function may be abnormally activated in liver diseases, exacerbating the central transmission of α-syn [110].

Evidence for the liver producing α-syn primarily comes from in vitro experiments and clinical sample analyses. Studies have detected α-syn mRNA and protein expression in the livers of normal rats, primarily localized to the mitochondria and endoplasmic reticulum of hepatocytes [111]. In vitro cultured human hepatocyte lines also show α-syn expression, which increases under hypoxic and oxidative stress conditions, suggesting that hepatic α-syn production may be regulated by pathological factors [112]. Chronic Mn exposure can induce the misfolding of α-syn, transforming it from a neuroprotective state to a neurotoxic one. This process constitutes a key pathogenic mechanism of Mn accumulation-mediated parkinsonism associated with acquired hepatocerebral degeneration (AHD), and is closely linked to impaired Mn excretion caused by liver cirrhosis [107]. The study found that α-syn accumulates in ballooning hepatocytes of patients with NASH (no cirrhotics) [113]; notably, NASH patients also exhibit neurological alterations, at least cognitive impairment, and possibly mild motor abnormalities. While α-syn accumulation in NASH livers implies a potential presence of this protein in cirrhotic patients, this inference cannot be definitively confirmed without further experimental validation.

More importantly, the liver can alter the pathological properties of α-syn through posttranslational modifications, making it more prone to aggregation and conferring stronger neurotoxicity. As a metabolically active organ, the liver possesses abundant modifying enzyme systems (e.g., kinases, glycosyltransferases, and ubiquitin ligases) that can perform various posttranslational modifications on α-syn [114]. α-Syn can stimulate protein kinase A (PKA)-catalyzed phosphorylation of Tau at the Ser^262^ site, while casein kinase 2 (CK2) is capable of catalyzing the phosphorylation of α-syn at the Ser^129^ site, and PD-associated α-syn mutations further amplify this effect [115,116]. In the study of rat HE model, it was observed that the soluble α-syn level in the cerebellum showed a decreasing trend, while the expression of insoluble α-syn increased [117]. In vitro experiments confirm that liver-derived phosphorylated α-syn is more prone to forming oligomers and exhibits greater toxicity to dopaminergic neurons compared to unphosphorylated α-syn.

Glycation is another important form of α-syn modification in the liver [118]. The hexosamine pathway is highly active in the liver, transferring glucose metabolites to lysine residues of proteins to form O-linked glycation modifications. Studies have found significant glycation modifications of α-syn in the livers of cirrhotic patients, whereas glycated α-syn levels are extremely low in normal livers [119]. Glycation alters the spatial conformation of α-syn, enhances its binding capacity to cell membranes, and inhibits its degradation by proteasomes, leading to intracellular accumulation of α-syn [120]. Additionally, glycated α-syn can activate inflammatory responses in the liver, promoting the release of inflammatory cytokines. These cytokines can circulate to the CNS, activating microglia and creating an inflammatory microenvironment that facilitates the central transmission of α-syn [121].

Liver-modified α-syn can affect the CNS through multiple pathways. On one hand, modified α-syn can enter the CNS by crossing the BBB. Glycation and phosphorylation modifications enhance its binding capacity to receptors on endothelial cells of the BBB, promoting its uptake and transport into the CNS [122]. In a rat model with portosystemic shunts, injection of liver-derived glycated α-syn resulted in higher levels of α-syn entering the CNS compared to unmodified α-syn, along with increased α-syn aggregates in the substantia nigra [123]. On the other hand, liver-modified α-syn can be transported to the CNS via peripheral immune cells (e.g., monocytes and macrophages). These immune cells can phagocytose peripherally circulating α-syn, cross the BBB through “cell migration” pathways, and release α-syn aggregates, inducing aggregation of endogenous α-syn [84]. Studies show that α-syn levels are elevated in peripheral blood monocytes of cirrhotic patients, and these cells exhibit an enhanced ability to cross the BBB, suggesting that immune cells may serve as “carriers” for the hepatic transmission of α-syn to the CNS [124].

#### Dysfunction of the vagus nerve

As the core neural pathway of the gut–liver–brain axis, the functional integrity of the vagus nerve not only determines the efficiency of signal transmission between the gut and the CNS but also directly influences the process of gut-to-central transmission of α-syn. Under liver disease conditions, chronic inflammation and metabolic disorders (such as hyperammonemia and energy imbalance) disrupt the vagus nerve both structurally and functionally, leading to reduced vagal tone and impaired conduction. This alters its characteristics as a pathological transmission pathway—shifting from “restricted transmission” under normal conditions to “permissive transmission” under pathological conditions—thereby accelerating the diffusion of α-syn to the CNS. This process can be understood through a progressive mechanism of “structural damage–functional impairment–altered transmission properties” [125].

In terms of structural damage, chronic inflammation associated with liver disease directly causes degeneration of vagus nerve fibers and damage to the myelin sheath. Patients with cirrhosis or NAFLD often experience gut dysbiosis, where overgrowth of Gram-negative bacteria in the intestine releases LPS. Impaired liver function prevents effective clearance of LPS, leading to systemic chronic inflammation upon entry into the systemic circulation [126]. These inflammatory cytokines can act on the vagus nerve (particularly the nodose ganglion and nerve fibers) via the bloodstream, activating local microglia and macrophages to release proinflammatory mediators and ROS, resulting in nerve fiber damage. In a rat model of cirrhosis, the myelin sheath thickness of vagus nerve fibers is reduced compared to normal rats, with vacuolar degeneration observed in axons and a higher rate of demyelination. Transmission electron microscopy reveals that inflammatory cytokines disrupt the tight junction structure of the myelin sheath, causing layering and fragmentation. The myelin sheath is critical for rapid nerve signal conduction, and its damage directly impairs the conduction function of the vagus nerve [127]. Additionally, inflammation inhibits the survival of vagus nerve neurons. In vitro studies show that LPS treatment reduces the survival rate of cultured nodose ganglion neurons and increases the activity of apoptosis-related proteins, suggesting that inflammation can reduce the number of vagus nerve neurons by inducing apoptosis, further weakening its function [128].

Metabolic disorders associated with liver disease (such as hyperammonemia and energy metabolism abnormalities) exacerbate vagus nerve dysfunction, leading to reduced vagal tone. Hyperammonemia is a core pathological feature of HE, and ammonia affects vagus nerve function through multiple pathways: On one hand, ammonia directly inhibits the electrical activity of vagus nerve neurons. In vitro patch-clamp experiments show that ammonia treatment reduces the firing frequency of action potentials in vagus nerve neurons and decreases the absolute value of the resting membrane potential, leading to reduced neuronal excitability [129]. On the other hand, ammonia inhibits the activity of choline acetyltransferase in vagus nerve neurons, a key enzyme for synthesizing the neurotransmitter acetylcholine. Reduced activity of this enzyme decreases acetylcholine synthesis, and since acetylcholine is the primary excitatory neurotransmitter of the vagus nerve, its reduction directly lowers vagal tone [130]. Clinical studies have found that cirrhotic patients with HE exhibit significantly lower vagal tone compared to healthy individuals, and the degree of vagal tone reduction is negatively correlated with blood ammonia levels, confirming the association between hyperammonemia and reduced vagal tone [131]. Furthermore, energy metabolism abnormalities caused by liver disease also affect vagus nerve function. The liver is a central organ for energy metabolism, and impaired liver function disrupts glucose and fatty acid metabolism, leading to insufficient energy supply for vagus nerve neurons [132]. Both electrical activity and neurotransmitter synthesis in neurons require substantial energy, and energy deficiency further suppresses vagus nerve excitability, exacerbating the reduction in vagal tone.

Structural damage and functional impairment of the vagus nerve alter its characteristics as a pathway for α-syn pathological transmission, shifting from “inhibiting transmission” to “promoting transmission”. Under normal conditions, the vagus nerve employs a “selective conduction” mechanism, allowing only physiological signals (such as those related to intestinal motility and digestive secretion) to pass while acting as a barrier to pathological substances (such as α-syn aggregates). This barrier function primarily relies on the myelin sheath structure of vagus nerve fibers and the protein degradation systems within neurons [133]. However, under liver disease conditions, damage to the myelin sheath of the vagus nerve compromises its barrier function, allowing α-syn aggregates to rapidly diffuse through demyelinated nerve fibers [134]. Simultaneously, the protein degradation systems within vagus nerve neurons become impaired due to energy deficiency and inflammatory damage, failing to effectively clear incoming α-syn. This leads to the accumulation of α-syn within neurons, forming “seeds” that induce aggregation of endogenous α-syn [135].

Animal experiments further confirm the role of vagus nerve dysfunction in promoting α-syn transmission. In a rat model with portosystemic shunts, intestinal injection of fluorescently labeled α-syn fibrils resulted in faster α-syn transmission in the vagus nerve dysfunction group compared to the normal group. The time to appearance of α-syn aggregates in the substantia nigra was shortened, and the loss of dopaminergic neurons in the substantia nigra increased [28,136]. Conversely, electrical stimulation of the vagus nerve or administration of acetylcholine receptor agonists slowed α-syn transmission and reduced the content of α-syn aggregates in the substantia nigra, suggesting that improving vagus nerve function can inhibit α-syn transmission [137]. Clinical studies have also found that cirrhotic patients with vagus nerve dysfunction have a significantly higher incidence of PD-like symptoms compared to those with normal vagus nerve function, along with significantly worse motor function scores. This confirms the clinical association between vagus nerve dysfunction and α-syn transmission, as well as PD-like symptoms [59].

## Other Synergistic Molecular Mechanisms

**Fig. 5.**
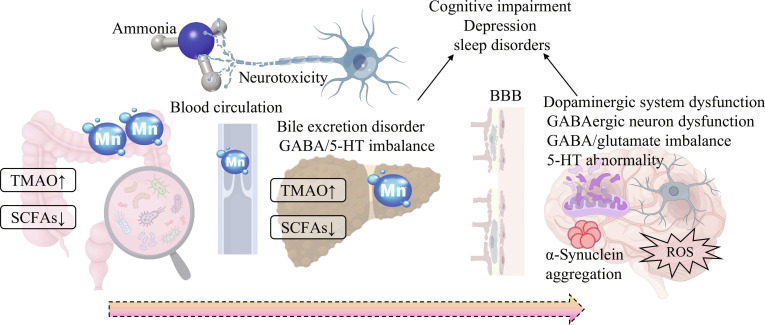
The multi-molecular synergistic mechanism linking HE and PD. Gut microbiota dysbiosis leads to a reduction in SCFAs and an increase in TMAO, while liver disease causes impaired bile excretion, resulting in Mn accumulation. Ammonia has direct neurotoxicity, while Mn, TMAO, and abnormal gut metabolites can affect the CNS through pathways such as blood circulation, combined with imbalances in GABA/5-HT. These factors can disrupt the BBB, leading to dopaminergic system dysfunction, impaired GABAergic neuron function, GABA/glutamate imbalance, and 5-HT abnormalities. They also promote α-syn aggregation, ROS generation, mitochondrial dysfunction, and microglial activation, ultimately causing nonmotor symptoms common to both HE and PD, such as cognitive impairment, depression, and sleep disorders. Together, these factors form a multi-molecular network connecting the 2 diseases [145,149,163,169].

In addition to the direct neurotoxicity of ammonia and the α-syn propagation regulated by the gut–liver–brain axis, there are multiple synergistic molecular mechanisms in liver disease states that exacerbate neural damage through independent or combined effects, promoting the occurrence of PD-like symptoms in HE patients and increasing the risk of PD onset. These mechanisms include basal ganglia damage caused by Mn deposition, motor and nonmotor symptoms induced by neurotransmitter system disorders, and the regulation of neuroinflammation and α-syn aggregation by abnormal gut microbiota metabolites. Together with the core mechanisms (ammonia, gut–liver–brain axis), they form a “multimolecular network” linking HE and PD, explaining the pathological association between the 2 diseases from multiple dimensions (Fig. 5).

### Mn deposition

Mn is an essential trace element in the human body, involved in the function of various enzymes and energy metabolism. Under normal physiological conditions, Mn is mainly absorbed through the intestine, enters the liver via the portal vein, and is then excreted back into the intestine through bile, forming a balanced “absorption–excretion” cycle [138]. However, in liver disease states such as cirrhosis and cholestatic liver disease, the liver’s bile excretion function is impaired, preventing Mn from being effectively excreted from the body. As a result, Mn accumulates in the body and crosses the BBB into the CNS, preferentially depositing in the basal ganglia (especially the globus pallidus) and causing manganism-induced encephalopathy. Its clinical symptoms and imaging findings are highly similar to those of PD, making it an important cause of parkinsonism-like symptoms in HE patients [139].

The core mechanism underlying Mn deposition in liver disease is bile excretion disorder. Under normal circumstances, Mn in the liver binds to glutathione to form complexes, which are then transported into bile for excretion via relevant transporters. In cirrhotic patients, massive hepatocyte necrosis and destruction of hepatic lobule structure lead to decreased expression of these transporters, reducing the liver’s ability to excrete Mn into bile [140]. In patients with cholestatic liver disease, bile duct obstruction blocks the bile excretion pathway, further exacerbating Mn excretion disorders, resulting in Mn accumulation in organs such as the liver and a subsequent increase in plasma Mn concentration [141]. Accumulated Mn is actively taken up into the CNS through specific receptors and transporters on the BBB endothelial cells. Due to the high permeability of the BBB in the basal ganglia (especially the globus pallidus) and the abundance of relevant uptake carriers, these regions become the main sites of Mn deposition. The putamen and caudate nucleus also show some degree of deposition, while deposition in areas such as the cerebral cortex and hippocampus is insignificant [142]. Mn deposition in the basal ganglia has distinct imaging features: Symmetric hyperintensities in the globus pallidus can be observed on T1-weighted magnetic resonance imaging. Most cirrhotic patients with HE exhibit this finding, and the signal intensity is positively correlated with plasma Mn concentration and the severity of parkinsonism-like symptoms [143].

Mn deposition induces parkinsonism-like symptoms through multiple mechanisms. First, Mn directly damages the nigrostriatal dopaminergic system by inhibiting the activity of tyrosine hydroxylase and aromatic L-amino acid decarboxylase (AADC)—key enzymes involved in dopamine synthesis, reducing dopamine production, and inducing apoptosis of nigral dopaminergic neurons [144]. Second, Mn disrupts the γ-aminobutyric acid (GABA)ergic neural circuits in the basal ganglia, leading to degeneration of related neurons, reduced neurotransmitter synthesis, and imbalance in motor regulatory circuits, resulting in symptoms such as muscle rigidity and bradykinesia [145]. Additionally, Mn can activate microglia to release inflammatory factors, further inhibiting the activity of tyrosine hydroxylase and promoting the death of dopaminergic neurons, forming a vicious cycle [146].

Clinical studies have confirmed the association between Mn deposition and parkinsonism-like symptoms. Cirrhotic patients with globus pallidus Mn deposition have a significantly higher incidence of parkinsonism-like symptoms than those without deposition, with muscle rigidity and bradykinesia being the main manifestations and resting tremor occurring less frequently [147]. After reducing plasma Mn concentration through chelator therapy, some patients show improved symptoms and reduced hyperintensity in the globus pallidus, indicating that Mn deposition is a reversible cause of parkinsonism-like symptoms in HE patients [148].

### Neurotransmitter system disorders: Associations between GABA/glutamate imbalance, 5-hydroxytryptamine changes, and nonmotor symptoms

Although HE and PD belong to different systemic diseases, their nonmotor symptoms overlap significantly. The incidence of depression, cognitive impairment, and other symptoms is much higher than in the general population, seriously affecting patients’ quality of life. Traditional studies have mostly focused on the impact of the dopamine system on motor symptoms while neglecting the core role of other neurotransmitter disorders in nonmotor symptoms. In fact, the dysregulated balance of GABA and glutamate in HE, and the concomitant reduction of both GABA and glutamate in PD are key triggers of nonmotor symptoms in both diseases and serve as important molecular links connecting HE and PD [147].

#### GABA/glutamate imbalance: Regulatory drugs and cross-talk with dopaminergic system

Cognitive impairment is one of the most prominent nonmotor symptoms in both HE and PD, characterized by decreased executive function, inattention, and memory loss in both. The core mechanism involves distinct patterns of GABA/glutamate dysregulation in the 2 diseases: In HE, ammonia accumulation disrupts the dynamic balance of GABA and glutamate [16]. Specifically, ammonia induces an imbalanced change in GABA relative to glutamate: Ammonia inhibits the activity of excitatory amino acid transporter 1/2 (EAAT1/2), reducing the reuptake of glutamate in the synaptic cleft and causing a mild, transient increase in synaptic glutamate levels [149]; ammonia significantly up-regulates the activity of glutamine synthetase in astrocytes, which diverts glutamate to glutamine synthesis, reduces the amount of glutamate available for neuronal release, and activates glutamic acid decarboxylase (GAD) to promote GABA synthesis and increase the sensitivity of GABA_A receptors [149]. This leads to a marked shift in the GABA/glutamate ratio toward GABA dominance in the brain, consistent with cerebrospinal fluid analyses of HE patients [150], which exacerbates central inhibitory tone and impairs hippocampal synaptic plasticity (e.g., blunted long-term potentiation)—directly underlying cognitive deficits such as memory loss and inattention. Drugs that regulate this balance in HE include benzodiazepine antagonists like flumazenil, which competes with GABA for binding to GABA_A receptors to reverse excessive central inhibition [151]; low-dose *N*-methyl-d-aspartate (NMDA) receptor modulators such as memantine, which moderates mild overactivation of NMDA receptors by synaptic glutamate [152]; and l-ornithine l-aspartate (LOLA), which restores astrocyte function to normalize glutamate reuptake while lowering ammonia [153].

In PD, by contrast, the pathological hallmark of GABA/glutamate dysregulation is a concomitant reduction in both neurotransmitters in brain regions critical for cognition (e.g., hippocampus and temporal cortex). Lewy body deposition in these regions causes degeneration of glutamatergic pyramidal neurons (reducing glutamate release) and loss of GABAergic interneurons (decreasing GABA levels) [154]. Unlike HE, the GABA/glutamate ratio in PD remains relatively close to physiological levels (due to parallel reductions in both neurotransmitters), but the absolute depletion of both signaling molecules disrupts the excitatory–inhibitory (E/I) balance in neural circuits [155]. This disruption impairs information processing and neural network coordination, leading to cognitive decline—mirroring the cognitive deficits in HE but via a distinct molecular pattern. Notably, both HE and PD patients show improved cognitive function with therapies targeting E/I balance, for example, low-dose NMDA receptor modulators for HE and GABAergic interneuron protectants for PD, confirming that GABA/glutamate dysregulation (via different patterns) is a shared pathological basis for cognitive impairment in both conditions [156]. Drugs targeting this balance in PD include GABAergic enhancers like baclofen, which compensates for GABA depletion in the basal ganglia to alleviate dystonia [156]; glutamate receptor antagonists such as amantadine, which inhibits excessive glutamate-mediated excitatory transmission to improve levodopa-induced dyskinesia [157]; and neurotrophic factors like brain-derived neurotrophic factor (BDNF), which protects GABAergic interneurons to restore E/I balance [158].

Notably, GABA and glutamate are key regulators of dopaminergic neuron activity in the substantia nigra and striatum, and their dysregulation in HE directly induces dopaminergic alterations—this is a core pathological link between HE and PD. Under normal conditions, glutamatergic inputs from the cortex and thalamus activate dopaminergic neurons in the substantia nigra pars compacta via NMDA receptors, while GABAergic inputs from the striatum and globus pallidus inhibit these neurons via GABA_A/B receptors, maintaining normal dopamine release in the striatum [159]. In HE, the GABA-dominant ratio disrupts this balance: Excessive GABAergic inhibition and blunted glutamatergic excitation synergistically suppress substantia nigra dopaminergic neuron activity, reducing striatal dopamine release [160]. In patients with HE, dopamine levels in the striatum are reduced and the expression of tyrosine hydroxylase, the rate limiting enzyme for dopamine synthesis, is decreased in neurons in the substantia nigra pars compacta [161], which explains why HE patients exhibit Parkinson's like motor symptoms such as bradykinesia and muscle rigidity. In PD, the concomitant depletion of GABA and glutamate further exacerbates dopamine deficiency: Reduced glutamatergic excitation fails to activate residual substantia nigra pars compacta neurons, while reduced GABAergic inhibition loses its “fine-tuning” role for dopamine release, worsening the motor symptoms caused by substantia nigra pars compacta degeneration [162].

#### 5-Hydroxytryptamine synthesis: Link with tryptophan entry into the brain

Depression and sleep disorders are the most distressing nonmotor symptoms in HE and PD patients, and these are closely related to abnormal 5-hydroxytryptamine (5-HT) system function—with 5-HT synthesis strictly dependent on tryptophan (Trp) entry into the brain, a process disrupted in both diseases [163]. The normal process of 5-HT synthesis follows a clear sequence: Trp is absorbed from the diet into the peripheral circulation, where most binds to albumin and a small portion remains free [164]; free Trp competes with other large neutral amino acids (LNAA) for binding to the L-type amino acid transporter 1 (LAT1) on the BBB—only Trp that successfully binds to LAT1 can cross the BBB, making this the rate-limiting step for central 5-HT synthesis [165]; once in the brain, Trp is taken up by serotonergic neurons in the raphe nucleus and converted to 5-hydroxytryptophan (5-HTP) by tryptophan hydroxylase 2 (TPH2; the rate-limiting enzyme for 5-HT synthesis); 5-HTP is rapidly converted to 5-HT by AADC, which is then stored in synaptic vesicles and released to regulate emotion and sleep [166].

In HE, ammonia disrupts this process at multiple steps: It increases the ratio of LNAA to Trp in the peripheral circulation and up-regulates LAT1 expression on the BBB, enhancing LNAA competition and reducing Trp entry into the brain [165]; it induces mitochondrial dysfunction and oxidative stress to inhibit TPH2 activity, slowing 5-HT synthesis even when Trp is available [167]; it activates monoamine oxidase A (MAO-A) to accelerate 5-HT degradation; and it down-regulates the expression of 5-HT1A/2A receptors, weakening 5-HT’s regulatory effects [42]. In PD, Lewy body deposition in the raphe nucleus causes degeneration of serotonergic neurons, reducing Trp uptake and TPH2 expression to disrupt the same pathway [168]. Clinical studies confirm significantly reduced cerebrospinal fluid 5-HT concentrations in both HE and PD patients (negatively correlated with depression and sleep disturbance scores), and both groups respond to selective 5-HT reuptake inhibitors (SSRIs) such as fluoxetine—these drugs block 5-HT reuptake in the synaptic cleft to compensate for reduced synthesis [169].

It is worth noting that neurotransmitter disorders in HE and PD interact synergistically: In HE, the GABA-dominant GABA/glutamate ratio further suppresses the activity of serotonergic neurons in the raphe nucleus (via enhanced GABAergic inhibition), exacerbating 5-HT deficiency; in PD, the combined loss of GABA and glutamate impairs the regulatory input to serotonergic circuits, forming a “neurotransmitter disorder cascade”. This cascade explains why nonmotor symptoms often co-occur and worsen in both diseases. Therefore, targeted regulation of the distinct GABA/glutamate dysregulation patterns (correcting the GABA-dominant ratio in HE, replenishing depleted GABA and glutamate in PD) combined with 5-HT system modulation may become a new direction for improving nonmotor symptoms in both conditions.

### Pathological associations between gut microbiota metabolites and liver disease–PD

Metabolites such as SCFAs and trimethylamine N-oxide (TMAO), produced by gut microbiota through metabolizing substrates like dietary fiber and choline, are key signaling molecules in the gut–liver–brain axis. In liver disease states such as cirrhosis and NAFLD, intestinal dysbiosis (decreased Bacteroidetes, increased Proteobacteria) disrupts metabolite balance, with the most significant changes being reduced SCFAs and increased TMAO. These 2 metabolites regulate neuroinflammation and interfere with α-syn aggregation, serving as important links connecting the pathological processes of liver disease and PD [170].

SCFAs (primarily acetate, propionate, and butyrate) are products of dietary fiber fermentation by SCFA-producing gut bacteria (e.g., Bifidobacterium and Faecalibacterium) and exert neuroprotective effects such as anti-inflammation and maintenance of BBB integrity [171]. In liver disease, the core cause of reduced SCFAs is microbial community imbalance: Cirrhotic patients have fewer SCFA-producing bacteria and more endotoxin-producing bacteria in the gut, leading to decreased total fecal SCFA concentration, with the most pronounced reduction in butyrate, which has the strongest neuroprotective effect [172]. Additionally, insufficient dietary fiber intake in liver disease patients further reduces substrates for SCFA synthesis.

Reduced SCFAs exacerbate neuroinflammation through dual pathways: On one hand, butyrate can activate microglial receptors to inhibit inflammatory pathways and reduce inflammatory factor release, whereas SCFA deficiency increases microglial activation and elevates inflammatory factor levels [173]; on the other hand, decreased SCFAs down-regulate the expression of tight junction proteins in the BBB, increasing endotoxin entry into the brain and further activating microglia, forming an inflammatory cycle [174]. Regarding α-syn aggregation, SCFAs (especially butyrate) promote α-syn acetylation by inhibiting the activity of histone deacetylases (HDACs), particularly HDAC6, enhancing its solubility [175]. In liver disease, reduced SCFAs lead to increased activity of HDACs, decreased α-syn acetylation, and elevated oligomer content. Animal experiments confirmed that supplementing butyrate in cirrhotic rats reduced inflammatory factors in the substantia nigra, decreased α-syn oligomers, and reduced dopaminergic neuron loss, verifying the pathological role of SCFA reduction [176].

TMAO is produced by gut microbiota metabolism of choline and carnitine [gut trimethylamine (TMA)-producing bacteria convert substrates to TMA, which is then oxidized to TMAO by liver enzymes]. The mechanisms underlying increased TMAO in liver disease are 2-fold: First, the number of TMA-producing bacteria increases, and high-protein diets in patients augment substrate intake, leading to increased TMA production [177]; second, liver inflammation activates related pathways, increasing the activity of converting enzymes to enhance TMA-to-TMAO conversion efficiency, while decreased liver function reduces TMAO clearance. Ultimately, plasma TMAO concentration increases and positively correlates with HE severity [178].

The neurotoxicity of TMAO manifests in 3 aspects: First, TMAO disrupts mitochondrial membrane potential in dopaminergic neurons, inhibits respiratory chain complex activity, increases ROS production, and suppresses antioxidant enzyme activity, exacerbating oxidative stress damage [179]; second, TMAO activates microglial pathways to promote inflammatory factor release, aggravating neuroinflammation [180]; most importantly, TMAO alters α-syn surface charge, enhances intermolecular hydrophobic interactions, accelerates oligomerization, increases fibrillation, and inhibits autophagic clearance, leading to accumulation of α-syn aggregates [19]. Animal experiments showed that mice injected with TMAO had increased α-syn oligomers in the substantia nigra, dopaminergic neuron loss, and PD-like symptoms; conversely, using TMAO inhibitors in cirrhotic mice reduced plasma TMAO, decreased α-syn aggregates, and significantly improved HE symptoms, confirming TMAO’s key role in liver disease-related neural damage [19].

## Translational Medicine and Prospects for New Therapeutic Strategies

Research on the pathological association mechanisms between HE and PD ultimately needs to be translated into clinical practice—achieving risk early warning by identifying diagnostic biomarkers and developing new therapeutic strategies targeting core mechanisms to improve PD-like symptoms in HE patients and reduce the risk of PD onset. Based on the mechanisms such as ammonia toxicity, gut–liver–brain axis disorder, and neurotransmitter imbalance elaborated above, this section prospects the application directions and prospects of translational medicine from 2 aspects: screening of diagnostic biomarkers and development of new therapeutic targets.

### Diagnostic biomarkers: Searching for biomarker associated with both liver dysfunction and neurodegeneration risk

Traditional diagnosis of HE relies on blood ammonia levels and clinical symptoms, while PD diagnosis is based on motor symptoms and imaging examinations, but both are difficult to identify PD risks related to HE at an early stage. An ideal biomarker should reflect both liver dysfunction and neurodegenerative tendency, and improve sensitivity and specificity through a combination of multiple indicators. Current research mainly focuses on 3 types of biomarkers.

The first type is “metabolic-inflammatory” composite biomarkers. Although blood ammonia is a core metabolic indicator for HE, its value in predicting PD risk alone is limited; combined detection with inflammatory factors such as IL-1β and TNF-α can significantly improve the predictive efficacy. Clinical studies have shown that patients with simultaneous abnormalities in blood ammonia and specific inflammatory factors have a significantly higher incidence of PD-like symptoms and risk of onset [181]. In addition, the combination of liver function indicators and neuroinflammatory markers also helps to distinguish between simple HE patients and those with neurodegenerative risk [182].

The second type is α-syn subtypes in exosomes. Exosomes can cross the BBB and enter the peripheral circulation, and the α-syn oligomers they carry are early markers of neurodegeneration. Studies have shown that the level of α-syn oligomers in plasma exosomes of HE patients is significantly increased, and it is positively correlated with the degree of liver function damage [183]. This indicator has high value in predicting the long-term risk of PD onset, and its performance is better than that of single blood ammonia detection. If phosphorylated α-syn and liver-specific proteins are simultaneously detected in exosomes, the specificity in judging the risk of “hepatic-derived” neurodegeneration can be further improved.

The third type is gut microbiota metabolite biomarkers. The concentration ratio of SCFAs to TMAO can reflect both gut microbiota imbalance and neurotoxic risk [184]. This ratio is significantly reduced in HE patients, and those with a ratio below a certain threshold show more significant neuroinflammation levels and a tendency for α-syn aggregation. Further clinical follow-up evidence suggests that this ratio can be used as a dynamic monitoring indicator, and patients with a lower ratio have a faster progression of PD-like symptoms.

### New therapeutic targets: Multidimensional interventions to reduce the risk of hepatogenic neurodegeneration

Based on the association mechanisms between HE and PD, therapeutic strategies need to go beyond the traditional single ammonia-lowering model and shift toward multi-target comprehensive interventions that integrate liver function repair, neuroprotection, and regulation of the gut–liver–brain axis. Below is a detailed analysis of the advantages, disadvantages, and clinical prioritization of core therapeutic strategies.

#### “Ammonia clearance +” combination therapy

Ammonia is the core neurotoxic factor linking HE and PD, so ammonia-lowering therapy serves as the foundation of intervention. The key advantage of this combination strategy lies in its ability to target multiple links in the formation of the “neurotoxic soil” by pairing ammonia-lowering drugs with anti-inflammatory agents, α-syn aggregation inhibitors, or neuroprotective drugs. For instance, combining lactulose or ornithine aspartate with minocycline not only reduces ammonia levels but also inhibits microglial activation, achieving synergistic therapeutic effects [185]. Additionally, GLP-1 receptor agonists like exenatide can simultaneously improve liver insulin resistance and protect dopaminergic neurons, aligning with the systemic pathological characteristics of the HE-PD association [186].

However, this approach has notable limitations. Long-term use of ammonia-lowering drugs may lead to side effects such as intestinal flora disturbance or electrolyte imbalance. Nonselective anti-inflammatory drugs (e.g., minocycline) lack sufficient specificity and may interfere with normal immune function. Moreover, some targeted drugs (e.g., nilotinib) have limited clinical data regarding their use in HE-related neurodegeneration, and their safety and efficacy in patients with liver impairment still require verification. In terms of clinical prioritization, “ammonia clearance +” combination therapy is recommended as a first-line intervention for HE patients with elevated blood ammonia and obvious neuroinflammation (e.g., increased IL-1β or TNF-α levels). Combinations with proven safety in liver disease populations—such as lactulose + minocycline or ornithine aspartate + exenatide—should be prioritized. For patients with mild hyperammonemia but a high risk of α-syn aggregation, nilotinib may be added under close monitoring of liver function [187].

#### Gut–liver–brain axis regulation strategy

##### Intestinal regulation

Intestinal regulation targets the source of pathological factors—gut microbiota imbalance—and boasts good safety and tolerability. Probiotics (e.g., Bifidobacterium and Faecalibacterium) can increase the production of SCFAs and reduce TMAO levels, fundamentally improving the neurotoxic microenvironment [188]. For severe gut dysbiosis, fecal microbiota transplantation (FMT) exhibits significant effects: It can quickly reverse intestinal barrier damage and reduce LPS translocation into the systemic circulation [189]. Yet, this strategy faces challenges. The efficacy of probiotics is strain-specific, with large individual differences, and long-term adherence is required to maintain benefits. FMT carries potential risks of infection transmission, and optimal donor screening criteria and treatment courses have not yet been standardized. Additionally, the action cycle of intestinal regulation is long, making it unsuitable for acute exacerbations of HE-PD symptoms. Clinically, intestinal regulation is recommended as a basic intervention for chronic HE patients, especially those with intestinal dysfunction (e.g., diarrhea and constipation) or abnormal SCFA/TMAO ratios. FMT is reserved for patients with refractory HE combined with severe gut dysbiosis and obvious PD-like symptoms, but it should only be used after excluding contraindications such as infections [190].

##### Neural pathway intervention

Vagus nerve stimulation (VNS) directly targets the α-syn transmission pathway, enabling rapid improvement of vagus nerve conduction function and reduction of pathological protein spread [191]. It also regulates neurotransmitter balance (e.g., increasing acetylcholine release), which benefits both motor and nonmotor symptoms of HE-PD [192]. The main drawbacks of VNS include risks associated with invasive procedures (e.g., infection and nerve injury), while noninvasive VNS (e.g., transcutaneous auricular VNS) has relatively weak efficacy. Furthermore, the optimal stimulation parameters (frequency, intensity) for HE-PD patients have not been determined, and individual response differences are significant. In clinical practice, VNS is a second-line intervention for patients with confirmed vagus nerve dysfunction (e.g., reduced vagal tone) and progressive PD-like symptoms. Noninvasive VNS is preferred for initial trials; invasive VNS may be considered for patients with poor response to noninvasive methods and severe symptoms, but it requires evaluation by a multidisciplinary team (including neurology, gastroenterology, and surgery specialists).

#### Lifestyle interventions

In addition, lifestyle interventions are equally important, particularly targeting shared risk factors of HE and PD, such as NAFLD and metabolic syndrome. Specifically, adopting a low-protein, high-fiber diet can reduce the production of ammonia and TMAO in the intestine while promoting the synthesis of SCFAs [193]. Sustaining moderate-intensity aerobic exercise not only improves insulin resistance but also enhances the secretion of neurotrophic factors. These neurotrophic factors [e.g., BDNF and glial cell line-derived neurotrophic factor (GDNF)] not only protect neurons (beneficial for both HE and PD) but also reduce α-syn aggregation through multiple key pathways. On one hand, they facilitate α-syn clearance: One way is to promote autophagy and UPS-mediated degradation, which helps reduce α-syn oligomers [194]; another way is to restore the function of lysosomes, supporting the breakdown of α-syn aggregates [195]. On the other hand, they stabilize the normal structure of α-syn by up-regulating heat shock proteins (e.g., ??HSP70), which prevents the misfolding of α-syn and is beneficial for PD [196]. At the same time, they alleviate the triggering factors of α-syn aggregation, clear ROS and repair mitochondrial damage, and also protect mitochondria from α-syn abnormalities caused by energy deficiency [197]. They also regulate the signaling pathway to inactivate glycogen synthase kinase 3β (GSK3β), a kinase that promotes harmful modifications of α-syn and enhances synaptic protein expression to bind to α-syn, thereby reducing free α-syn monomers [198]. Furthermore, neurotrophic factors can act synergistically with GABA/glutamate regulators (e.g., enhancing the efficacy of LOLA in HE), making them promising targets for combined therapies.

Nonetheless, this strategy relies heavily on patient compliance, and its efficacy is slow to manifest (typically requiring 3 to 6 months to become apparent). For patients with severe liver dysfunction or advanced PD-like symptoms, lifestyle interventions have limited effects and cannot replace drug therapy. Clinically, lifestyle interventions are recommended as a universal basic measure for all HE patients, regardless of PD risk. They should be combined with drug or device-based therapies to enhance long-term efficacy. For patients with NAFLD-related HE, lifestyle interventions (diet adjustment + exercise) should be prioritized to delay liver function deterioration and reduce PD risk.

In summary, by combining biomarker early warning, multi-target drug combination therapy, and lifestyle interventions to form a complete translational medicine prevention and control pathway, it is expected to identify the neurodegeneration risk in HE patients at an early stage and provide effective interventions, opening up new directions for improving patient prognosis and reducing the incidence risk of PD.

## Conclusion and Future Directions

The research value of HE goes far beyond the scope of the disease itself. It provides a unique human pathological model for analyzing how environmental metabolic factors (such as ammonia accumulation, intestinal flora disorder, and liver dysfunction) drive neurodegenerative diseases (represented by PD). When in the HE state, the liver, as a “metabolic center”, fails to function, triggering a series of chain reactions such as ammonia toxicity, gut–liver–brain axis disorder, and neurotransmitter imbalance. Eventually, a “neurotoxic soil” is formed in the CNS—it may induce PD in susceptible individuals, accelerate the progression of subclinical PD, and also simulate PD-like pathology. This process is not the isolated action of a single molecule or system, but the result of the dynamic evolution and mutual coordination of multiple molecules such as ammonia, α-syn, and intestinal flora metabolites (SCFAs, TMAO), as well as multiple systems such as the intestine, liver, and brain. It provides a panoramic perspective for understanding the association between environmental metabolic factors and neurodegenerative diseases.

This review systematically elucidates the convergence of molecular mechanisms between HE and PD through pathways such as the gut–liver–brain axis, which not only updates the understanding of neurological complications in HE but also provides a new framework for comprehending the systemic origins of neurodegenerative diseases such as PD. Multi-target comprehensive intervention strategies proposed based on these mechanisms, including “ammonia-lowering+” and “gut–liver–brain axis modulation”, highlight the necessity of transitioning from single symptomatic treatment to systemic treatment targeting the “neurotoxic soil”. This provides an important theoretical basis and direction for clinically identifying the neurodegenerative risk in patients with liver diseases and formulating early intervention plans.

To deepen the understanding of the HE-PD association mechanisms and promote their clinical translation, future research needs to achieve breakthroughs at multiple levels. First, efforts should be made to establish dual-pathology animal models that more closely resemble human pathology, such as constructing “liver injury + α-syn overexpression” animal models to reproduce the dynamic continuum of “metabolic abnormalities–neurodegeneration” under HE conditions, thereby providing more reliable tools for mechanistic exploration and drug screening. Second, conducting large-scale, prospective clinical cohort studies is crucial. These studies should involve long-term follow-up of PD risk and changes in neuropathological biomarkers in patients with different types of liver diseases, aiming to identify key predictors of HE-related PD risk and validate the effectiveness of early interventions. Third, advanced technologies such as organoids and single-cell sequencing should be utilized to explore intercellular communication mechanisms in depth. For example, brain–liver organoid coculture systems can be employed to analyze the interactions among hepatocytes, neurons, and glial cells, while single-cell sequencing can be used to identify specific cell subpopulations and their molecular regulatory networks associated with “liver-originated” neurotoxicity in brain tissues of HE patients, providing precise evidence for discovering new therapeutic targets. Finally, the ultimate goal of research should lie in promoting translational medicine and the development of personalized treatment strategies. Future efforts must focus on exploring how to translate the above molecular mechanisms into clinically applicable biomarker panels and step-by-step treatment plans. This includes optimizing the drug combinations and treatment timing for “ammonia-lowering+” combination therapies, standardizing the clinical application criteria for gut–liver–brain axis modulation strategies (such as probiotic strain selection, indications for FMT, and VNS parameters), and ultimately developing personalized treatment pathways based on patients’ specific metabolic–inflammatory–protein pathological phenotypes. This will establish a comprehensive clinical prevention and treatment system ranging from risk early warning to multi-target interventions.

## Data Availability

All data and material generated or analyzed during this study are included in this published article.
